# Filariasis of the Breast in a Pregnant Woman Diagnosed by Fine-needle Aspiration Cytology: A Case Report

**DOI:** 10.1155/S106474499500072X

**Published:** 1995

**Authors:** Melanie Lau, Pamela Tauchi, Milton Kim, Francis Liu, Thomas Namiki

**Affiliations:** 1 Department of Obstetrics and Gynecology University of Hawaii John A. Burns School of Medicine Honolulu HI USA; 2 Department of Pathology University of Hawaii John A. Burns School of Medicine Honolulu HI USA; 3 The Queen's Medical Center Department of Surgery Kapiolani Women and Children's Medical Center University of Hawaii John A. Burns School of Medicine Honolulu HI USA; 4 Division of Infectious Disease Kaiser Permanente Medical Center University of Hawaii John A. Burns School of Medicine Honolulu HI USA; 5 The Queen's Medical Center 1301 Punchbowl Street Honolulu HI 96813 USA

## Abstract

*Background:* Despite increased immigration to the United States from endemic areas, the diagnosis of microfilariasis in this country remains infrequent. This disease may occasionally present as a breast mass, in the absence of other clinical findings.

*Case:* We report an unusual case of *Wuchereria bancrofti* diagnosed in a pregnant woman by breast fine-needle aspiration (FNA) and discuss the clinical implications of filariasis in pregnancy.

*Conclusion:* FNA is safe and reliable in pregnancy. Infants of mothers with breast filariasis should be monitored.


Infectious Diseases in Obstetrics and Gynecology 3:245–247 (1995)
© 1996 Wiley-Liss, Inc.

### Filariasis of the Breast in a Pregnant Woman Diagnosed by Fine-Needle Aspiration Cytology: A Case Report

**Melanie Lau, Pamela Tauchi, Milton Kim, Francis Liu, and Thomas Namiki**

*Departments of Obstetrics and Gynecology (M.L.) and Pathology (P.T., T.N.), The Queen's Medical Center, Department of Surgery, Kapiolani Women and Children's Medical Center (M.K.), and Division of Infectious Disease, Kaiser Permanente Medical Center (F.L.), University of Hawaii John A. Burns School of Medicine, Honolulu, HI*

ABSTRACT

***Background:*** **Despite increased immigration to the United States from endemic areas, the diagnosis of microfilariasis in this country remains infrequent. This disease may occasionally present as a breast mass, in the absence of other clinical findings.**

***Case:*** **We report an unusual case of *Wuchereria bancrofti* diagnosed in a pregnant woman by breast fine-needle aspiration (FNA) and discuss the clinical implications of filariasis in pregnancy.**

***Conclusion:*** **FNA is safe and reliable in pregnancy. Infants of mothers with breast filariasis should be monitored.** © 1996 Wiley-Liss, Inc.

KEY WORDS

Microfilariasis, breast mass, *Wuchereria bancrofti*, Bancroft's filariasis

A 28-year-old Samoan woman, $G_3P_{2002}$, in her 18th week of pregnancy presented to a hospital clinic in Honolulu, HI, in April 1992. The patient had immigrated from American Samoa approximately 1 year prior to presentation. Palpation of the left breast revealed a 5-mm subcutaneous nodule adjacent to the lower inner edge of the areola. The nodule was firm and nontender, and the overlying skin was unremarkable. There was no history of a nipple discharge and the axillary lymph nodes were not enlarged. Other than the pregnancy, there were no other findings on physical examination. The medical history was otherwise unremarkable, and the patient was asymptomatic.

A fine-needle aspiration (FNA) was performed using a 25-gauge, 5/8-in. needle attached to a 3-cc syringe. The aspirate was smeared onto a slide, air-dried, and stained with Diff-Quik. A diagnosis of Bancroft's filariasis (*Wuchereria bancrofti*) was made. The cytologic examination revealed numerous sheathed microfilariae with elongated terminal nuclei and a caudal space of 5–15 μm at the posterior end (Fig. 1). There were also scattered inflammatory cells consisting of neutrophils, histiocytes, and multinucleated giant cells. Because of the pregnancy, it was elected to postpone treatment until after delivery.

The patient delivered a healthy female infant in October 1992. The pathologic examination of the placenta revealed no significant histopathology; an

Address correspondence/reprint requests to Dr. Pamela Tauchi, Director of Cytopathology, The Queen's Medical Center, 1301 Punchbowl Street, Honolulu, HI 96813.

Received October 13, 1995
Accepted December 29, 1995

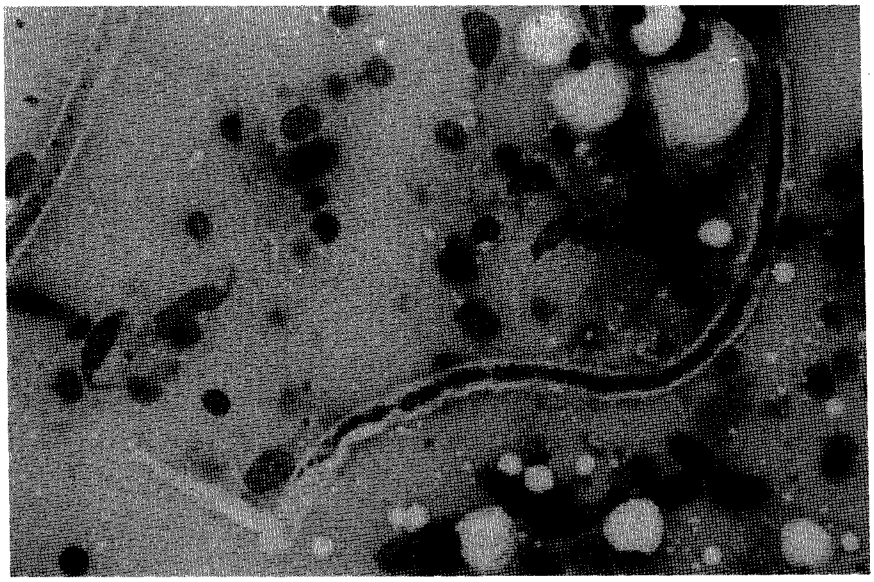

Fig. 1. Microfilaria of *W. bancrofti*. Diff-Quik stain, ×400.

examination of the cord blood was negative for microfilaria. After giving birth, the patient did not return for follow-up.

### DISCUSSION

Bancroft's filariasis has a worldwide distribution, with disease prevalence in Africa; Asia including China, India, and Southeast Asia; the Caribbean Islands; Central and South America; and the South Pacific. Most cases seen in the United States are those involving immigrants from these endemic areas.[1]

Insects, particularly mosquitoes, serve as intermediate hosts. While taking a blood meal, the insect ingests microfilariae. Over 2–3 weeks, the microfilariae develop within the insect into infective third-stage larvae. They reenter the definitive human host when the insect feeds again. The larvae mature into adult worms which live 10–15 years and produce microfilariae.[2]

The majority of those who are infected remain asymptomatic throughout life. Those patients with symptoms may manifest an acute phase of the disease characterized by fever and myalgias, lymphangitis, and lymphadenitis, with accompanying eosinophilia and microfilaremia. The chronic stage may be characterized by lymphadenopathy, lymphedema, and elephantiasis. The lymphatics of the lower extremities and genitalia are the most common sites of involvement, followed by the upper extremities. However, the organisms may be found in any organ of the body, provoking an inflammatory reaction and causing a mass lesion in the absence of the classic signs of filariasis.

Filarial infection of the breast is not uncommon, especially in endemic areas.[1] Microfilaria have been detected by the cytologic examination of a nipple discharge.[3] More recently, in endemic areas, FNA has been employed to diagnose cases of breast involvement.[4–7] Ours is the first reported case of microfilariasis diagnosed by FNA in the United States. Because of continued immigration from endemic areas, it is necessary to maintain awareness of this disease entity.

In the past, the definitive diagnosis of filariasis has often been based on an identification of the microfilariae in blood. The microfilariae of *W. bancrofti* often demonstrate periodicity, and the blood samples must be taken at night, preferably between 10 P.M. and 2 A.M. This periodicity may be absent in the South Pacific species, resulting in persistent microfilaremia. Nevertheless, newer studies have reported the cytologic diagnoses of filariasis by FNA from sites other than the breast such as the testis, epididymis, thyroid, lung, lymph node, and skin. A recent review[8] of these cases revealed that 18 of these patients had undergone blood examinations for microfilaria, of which only 2 (12%) were positive.

Therefore, because of the low yield and stringent sampling requirements of a blood examination, FNA cytology appears to be a more convenient and effective diagnostic method in patients with mass lesions.

In our case, the patient's state of pregnancy resulted in a unique situation with regard to management. There have been no reported cases of abortion associated with FNA of the breast.[9,10] Thus, when performed and interpreted by an experienced physician, FNA can be a safe and reliable method for evaluating breast masses in the pregnant patient.

The transplacental transmission of microfilaria to the fetus may occur in as many as 10% of pregnancies of infected mothers.[11] Although there have been no published reports of adverse effects to the fetus, it may be necessary to monitor the infants more closely for ensuing disease. Therefore, a pathologic examination of the placenta and an examination of the cord blood should be performed in all such cases.

### REFERENCES

1. Lang AP, Luchsinger IS, Rawling EG: Filariasis of the breast. Arch Pathol Lab Med 111:757–759, 1987.
2. Ottesen EA: Filarial infections. Infect Dis Clin N Am 7:619–633, 1993.
3. Lahiri VL: Microfilariae in nipple secretion. Acta Cytol 19:154, 1975.
4. Kapila K, Verma K: Gravid adult female worms of *Wuchereria bancrofti* in fine needle aspirates of soft tissue swelling: Report of three cases. Acta Cytol 33:390–392, 1989.
5. Bapat KC, Pandit AA: Filarial infection of the breast: Report of a case with diagnosis by fine needle aspiration cytology. Acta Cytol 36:505–506, 1992.
6. SenGupta SK, Webb S, Cooke RA, Igo JD: Breast filariasis diagnosed by needle aspiration cytology. Diagn Cytopathol 8:393–398, 1992.
7. Sodhani P, Murty DA, Pant CS: Microfilaria in a fine needle aspirate from a breast lump: A case report. Cytopathology 4:59–62, 1993.
8. Kaya B, Namiki T, Tauchi P: Cytologic diagnosis of bancroftian filariasis: Clinical implications. Acta Cytol 39:1042, 1995.
9. Byrd BF, Bayer DS, Robertson JC: Treatment of breast tumors associated with pregnancy and lactation. Ann Surg 155:940–947, 1962.
10. Bottles K, Taylor RN: Diagnosis of breast masses in pregnant and lactating women by aspiration cytology. Obstet Gynecol 66:76S–78S, 1985.
11. Eberhard JL, Hitch WL, McNeeley DF, Lammie PJ: Transplacental transmission of *Wuchereria bancrofti* in Haitian women. J Parasitol 79:62–66, 1993.
